# Return to work after subarachnoid hemorrhage: The influence of cognitive deficits

**DOI:** 10.1371/journal.pone.0220972

**Published:** 2019-08-09

**Authors:** Anne M. Buunk, Jacoba M. Spikman, Jan D. M. Metzemaekers, J. Marc C. van Dijk, Rob J. M. Groen

**Affiliations:** 1 Department of Neurology, Subdepartment of Neuropsychology, University of Groningen, University Medical Center Groningen, Groningen, the Netherlands; 2 Department of Neurosurgery, University of Groningen, University Medical Center Groningen, Groningen, the Netherlands; Universitatsklinikum Freiburg, GERMANY

## Abstract

**Introduction:**

Cognitive deficits are frequently found after subarachnoid hemorrhage (SAH), but their influence on return to work is largely unknown. To improve identification of those patients at-risk for long-term return to work problems, we aimed to examine the value of cognitive deficits in the prediction of long-term return to work after subarachnoid hemorrhage.

**Methods:**

SAH patients (N = 71) who were employed before SAH and were able to undergo neuropsychological assessment, were included. Demographic characteristics and acute SAH-related variables (SAH-type and external cerebrospinal fluid drainage) were taken into account. Neuropsychological tests for memory, speed, attention, executive function, and emotion recognition and a questionnaire for executive functions were used. Return to work was assessed using the Role Resumption List.

**Results:**

Results showed that patients with incomplete return to work had significantly lower scores on neuropsychological measures for complex attention and executive functions (*p* < 0.05) compared to patients with complete return to work. Return to work could not be significantly predicted using only demographic characteristics and acute SAH-related variables, but adding measures of complex attention and executive functions resulted in a prognostic model that could reliably distinguish between complete and incomplete return to work. Statistically significant predictors in the final model were cerebrospinal fluid drainage and scores on a questionnaire for executive functions: patients with cerebrospinal fluid drainage and higher scores on the a questionnaire for executive functions were less likely to return to work.

**Discussion:**

Together, these findings show that neuropsychological measures, especially for complex attention and executive functions, have added value to acute SAH-related and demographic variables in the prediction of long-term return to work after SAH.

## Introduction

Subarachnoid hemorrhage (SAH) is a severe subtype of stroke, that is associated with high morbidity and mortality. SAH often results from the rupture of an intracranial aneurysm (aneurysmal SAH [aSAH]), and no structural cause for the hemorrhage can be found in around 15% (angiographically negative SAH [anSAH]). Survivors often experience negative consequences that interfere with everyday life functioning [1]. Due to the losses of productive life years and the ability to participate socially and professionally, the effect on society is significant [2]. Previous research revealed that up to two-third of patients are unable to return to their former employment, even several years post-SAH [3–5]. Inability to return to work (RTW) is an important cause of dissatisfaction with life [6] and reduced quality of life [7]. Therefore, it seems relevant to explore which factors in an early stage allow to identify those patients at-risk for long-term RTW problems.

Previous search for predictors of RTW has been limited and focused mainly on acute SAH-related factors, such as SAH-type (aSAH or anSAH), severity, and complications (vasospasm, hydrocephalus). Results are ambiguous, some studies defined these clinical features as predictive for RTW post-SAH [8,9], while others did not [10,11]. Correlations have been found between post-SAH mood disorders and incomplete RTW [12,13], however, only one study identified depression as a predictor of RTW in a prognostic model [8].

Cognitive deficits are frequently found post-SAH, even in patients with good neurological outcome [1,14–17]. As everyday life is complex, with high demands being placed on the ability to make rapid and well-thought decisions, intact cognitive functioning is pivotal for SAH patients to resume and sustain their pre-SAH employment. The predictive value of cognitive deficits in the subacute phase for RTW in the long-term, has been well established in subgroups of stroke [18–20] and traumatic brain injury [21]. However, only a few studies have attempted to investigate the relationship between cognitive deficits and RTW after SAH. Those studies suggest a relationship between low performance on cognitive tests and a failure to RTW after aSAH [9,22]. Only two studies included cognitive measures in a prediction model of work status, and results are inconclusive. Wallmark and colleagues [23] used a global cognitive screening tool in their model and showed that RTW of 68% of the patients could be correctly predicted. In contrast, Vilkki et al. [11] concluded that several cognitive tests were of limited added value compared to high age, low education, and self-reported impairments. The aforementioned studies have focused on aSAH patients and consequently conclusions may not be applicable for both subtypes of SAH. Moreover, little is known about the specific influence of cognitive functions such as processing speed, attention, and social cognition on RTW, although it is known that these functions can also be impaired post-SAH [24–26].

The aim of the present study was twofold: (1) to investigate the association between cognitive functioning and RTW, and (2) to examine whether and which neuropsychological measures in the subacute stage (two to eight months post-SAH) had additional value to acute SAH-related variables and demographic characteristics, in the prediction of long-term RTW. This might allow early identification of patients who are at-risk for incomplete RTW and could lead to development of interventions to prevent RTW problems in the long-term.

## Materials and methods

### Patients and procedure

All survivors of SAH aged 18 years and older admitted to a University Medical Center between 2009 and 2013 were invited to participate in this study. Patients who were able to undergo neuropsychological assessment, who were employed before SAH and agreed to participate, were included. Exclusion criteria were insufficient proficiency of the Dutch language and serious co-morbidity. SAH was confirmed by means of a computed tomography (CT) scan in the acute stage. Using CT angiography and/or digital subtraction angiography at admission, the presence (aSAH) or absence (anSAH) of a symptomatic intracranial aneurysm was assessed. Patients received external (ventricular or lumbar) drainage of cerebrospinal fluid (CSF) for increased intracranial pressure, with or without enlarged ventricles on CT scan. In aSAH patients, location of the aneurysm and treatment (coiling/clipping) were documented. Data on clinical condition at admission (World Federation of Neurological Surgeons [27])were obtained from the patients’ medical reports.

Patients completed neuropsychological tests and questionnaires at approximately two to eight months post-SAH (time point 1, T1). In the chronic stage (between one and eight years post-SAH), changes in participation (vocational functioning, leisure activities, social interaction, and mobility) were investigated by a telephonic interview (time point 2, T2). For this study, changes in vocational functioning were used as the main outcome measure. Written informed consent was given by all participants. The study protocol was approved by the Medical Ethical Committee of the University Medical Center of Groningen (nr. 2009.164) and conforms to Helsinki Declaration.

### Demographic and acute SAH-related variables

Data on demographic and clinical variables were obtained from the patients' medical reports and are listed in Table 1. Educational level was scored on a 7-point scale from (1) = no primary school, to (7) university [28]. Aneurysm location was dichotomized in anterior (aneurysms of the anterior cerebral or communicating artery, middle cerebral artery, posterior communicating artery, internal carotid artery, ophthalmic artery, and anterior choroidal artery) and posterior (aneurysms of the basilar artery, posterior cerebral artery, superior cerebellar artery, and vertebral artery). Considering SAH-related variables, external CSF drainage was included in the regression analysis, since this has been related to unfavorable outcome in the literature [29,30]. Also, anSAH is generally considered a benign pathology compared to aSAH [31,32], therefore SAH-type was also entered.

**Table 1:** Patient characteristics.

	SAH patients (N = 71)
**Age at time of SAH (M ± SD)**	49.2 ± 7.9
**Sex (% female)**	60.6
**Educational level**	4.9 ± 1.1
** Low (1–4, %)**	33.8
** High (5–7, %))**	66.2
**Interval between SAH and T1 in months (M ± SD, range)**	4.6 ± 1.4, 2–8
**Interval between T1 and T2 in years (M ± SD, range)**	2.9 ± 1.6,1–8
** Between 1 and 4 years (%)**	84.5
** Between 5 and 8 years**	15.5
**WFNS**	1.0
** Low (1–3)**	84.5
** High (4–5)**	15.5
**External CSF drainage (yes, %)**	56.3
**Treatment aSAH**	
** Clipping (%)**	26.9
** Coiling (%)**	71.2
** None (%)**	1.9
Aneurysm circulation aSAH*	
** Anterior (%)**	88.5
** Posterior (%)**	11.5

SAH, subarachnoid hemorrhage; WFNS, World Federation of Neurological Surgeons; aSAH, aneurysmal subarachnoid hemorrhage. Educational level is scored on a 7-point scale from (1) = no primary school, to (7) university.

*Anterior: aneurysms of the anterior cerebral or communicating artery, middle cerebral artery, posterior communicating artery, internal carotid artery, ophthalmic artery, and anterior choroidal artery. Posterior: aneurysms of the basilar artery, posterior cerebral artery, superior cerebellar artery, and vertebral artery.

### Neuropsychological measures

#### Memory

The Dutch version of the Rey Auditory Verbal Learning Test (15 Words Test [15WT]) [33] was used to measure verbal memory. Participants are presented with a set of 15 words and are asked to reproduce as many of the words possible immediately after presentation, in 5 trials. The total number of words recalled in 5 trials is the total score, with a maximum of 75.

#### Information processing speed

The Trail Making Test (TMT [34]) consists of two parts and part A (TMT-A) was used to measure psychomotor speed. Participants are asked to connect 25 encircled numbers in ascending order, as quickly as possible. The total score represents the time in seconds to complete the task.

The Stroop Color-Word Test [35,36] consists of three subtests and the subtest Stroop Word was used to measure mental processing speed. Participants are instructed to read 100 color names printed in black ink and the total score is the time in seconds to complete the task.

#### Complex attention and executive functions

The Zoo Map test [37] was used to measure planning ability. Participants are required to show how they would visit specific locations on a map, while adhering to restricting rules. The maximum total score is 16.

The TMT part B (TMT-B) was used as measure of switching attention and cognitive flexibility [38]. Participants are required to connect 25 randomly placed encircled numbers and letters in ascending order, alternating between the two. Total score consists of the time in seconds needed to complete the test.

With the third subtest of the Stroop Color Word Test (Stroop Color-Word), participants are asked to name the colors of 100 color-words that are printed in an inconsistent color ink. In order to name the colors, the automatic tendency to read the words has to be repressed, therefore selective focusing and inhibition are measured [35,36,39].

#### Social cognition

The Facial Expression of Emotion-Stimuli and Test (FEEST [40]) is a measure of one aspect of social cognition: emotion recognition. Participants are shown sixty faces depicting the primary emotions (fear, disgust, happiness, sadness, anger, and surprise) and are asked to choose the correct emotion. Total scores range from 1 to 60.

#### Questionnaire for executive complaints

The Dysexecutive Questionnaire (DEX [37]) was used as a measure of executive impairment in daily life. The DEX consists of 20 items to be answered on a 5-point Likert scale. Maximum total score (DEX-Total) is 80 and a score above 27 indicates executive problems [41].

### Outcome measure

RTW was assessed using the Role Resumption List [42,43] in a telephonic interview and was scored in five categories: (0) no change, (1) previous work resumed with lower demands or part-time, (2) different work on a lower level, (3) working in a socially protected environment, and (4) not working. For analysis, scores were dichotomized in ‘complete RTW’ (0) and ‘incomplete RTW’ (1–4). Patients of 60 years and older were asked additional questions about retirement (e.g. “Were you planning to retire before the SAH?”, “Did you return to work after SAH, but before retiring?”), to ensure that the reason for incomplete RTW was related to the SAH (and not to retirement age).

### Statistical analysis

The data were analyzed using SPSS version 23.0 (SPSS Inc.). Descriptive statistics were used to describe the population demographic and clinical characteristics. Neuropsychological test performances were compared with normative data and performances below the tenth percentile or a cut-off score (FEEST, Zoo Map) were considered as clinically impaired [44]. To test for differences on neuropsychological measures between patients with complete and incomplete RTW, Mann-Whitney U tests were used. Effect sizes (Cohen’s *d*) were calculated for between-group comparisons. Independent relations between the possible predictors and RTW were tested using hierarchical binary logistic regression. Age, educational level, external CSF drainage, and SAH-type were entered in block 1. In block 2, variables were included on the basis of between-group comparisons; neuropsychological measures for which significant differences were found between patients with incomplete and complete RTW were entered. The overall alpha was set at 0.05 and Bonferroni-Holm corrections were applied in case of multiple comparisons.

## Results

### Participants

**Fig 1:**
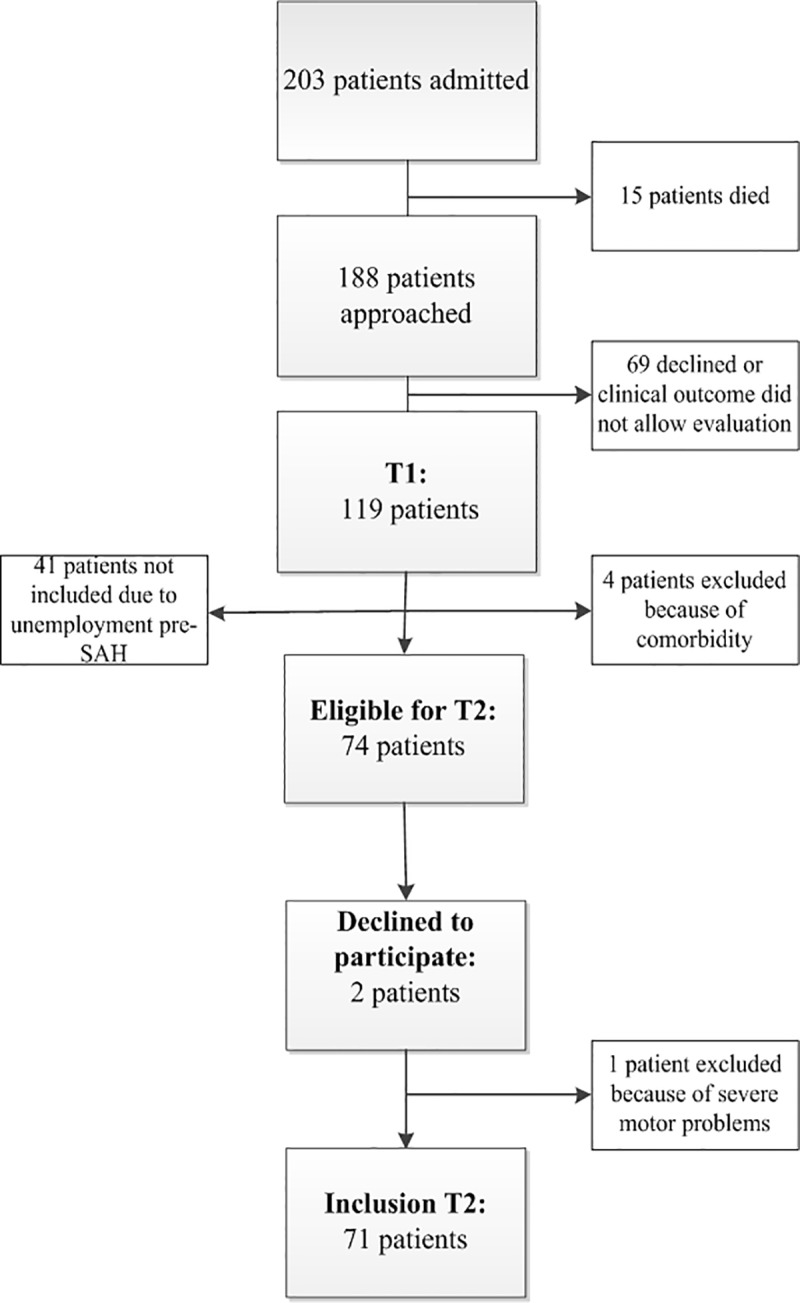
Flow chart of included and excluded patients.

The inclusion process for this study is illustrated in Fig 1. No significant differences were found between aSAH patients (N = 52) and anSAH patients (N = 19) with respect to age (M_aSAH_ = 49.7, SD_aSAH_ = 8.4, M_anSAH_ = 48.1, SD_anSAH_ = 6.5, t = 0.73, *p* = 0.47) and sex (percentage female 63.5% and 52.6% resp., χ^2^ = 0.68, *p* = 0.43). WFNS grade was significantly higher in aSAH patients (Mdn = 2.0) compared to anSAH patients (Mdn = 1.3), U = 823, *p* = 0.002) and the need for external CSF drainage was higher in aSAH (67.3%) compared to anSAH (26.3%), χ^2^ = 9.5, *p* = 0.003. Table 1 shows the characteristics of the included patients.

Table 2 shows that about one-third of all study participants reported complete return to previous employment between 1 and 8 years (M = 3.5, SD = 1.6) after SAH.

**Table 2:** Changes in vocational functioning as assessed by the Role Resumption List.

	N (total = 71)	Percentage
**No change**	24	33.8%
**Lower demands or part-time**	18	25.4%
**Different work, lower level**	2	2.8%
**Socially protected environment**	2	2.8%
**No return to work at all**	25	35.2%

### Neuropsychological measures and RTW

Impaired performances (<10^th^ percentile) were present in 45.1% of all patients on the memory task 15WT. For mental speed and attention, these percentages were 9.9% (TMT-A) and 31.0% (Stroop-Word) respectively. Deficits in executive functions and complex attention were evident in 9.9% (TMT-B), 14.1% (Stroop Color-Word), and 31.0% (Zoo Map) of all patients. FEEST total score (emotion recognition) was below cut-off in 23.9%. 76.1% of all patients had cognitive deficits, according to scores on one or more of the neuropsychological tests. Executive problems (DEX-Total > 27) were reported by 19.7% of all patients. Mean scores for the total patient group on all neuropsychological measures are listed in Table 3, as well as results regarding RTW and neuropsychological measures.

**Table 3:** Comparisons of neuropsychological measures between SAH patients with complete and incomplete RTW.

	All patients N = 71	Complete RTW N = 24	Incomplete RTW N = 47	U	p	Cohen’s *d*
**Neuropsychological tests**						
***Memory***						
**15WT**	40.1 ± 10.8	42.1 ± 12.3	39.1 ± 9.9	452.5	0.18	0.28
***Information processing speed***						
**TMT-A**	34.5 ± 13.8	29.6 ± 9.3	37.0 ± 15.2	398.0	0.04	0.56
**Stroop Word**	49.1 ± 10.6	49.0 ± 9.6	49.1 ± 11.2	562.5	0.99	0.01
***Complex attention and executive functions***						
**TMT-B**	78.7 ± 40.7	66.0 ± 32.6	85.2 ± 43.2	375.5	0.02*	0.49
**Stroop Color-Word**	97.1 ± 28.2	86.2 ± 19.9	102.7 ± 30.3	345.0	0.008*	0.61
**Zoo Map**	7.6 ± 5.8	9.3 ± 5.0	6.8 ± 6.0	445.5	0.15	0.45
***Social cognition***						
**FEEST**	46.3 ± 6.5	46.5 ± 6.5	46.3 ± 6.5	561.0	0.97	0.03
**Questionnaire**						
**DEX-Total**	16.1 ± 11.4	10.0 ± 6.9	19.2 ± 12.1	300.0	0.001*	0.88

SAH, subarachnoid hemorrhage; 15WT, 15 Words Test; TMT-A, Trail Making Test part A; TMT-B, Trail Making Test part B; DEX-Total, Dysexecutive Questionnaire total score; FEEST, Facial Expression of Emotion-Stimuli and Test; RTW, return to work.

* significant after Bonferroni-Holm correction.

After Bonferroni-Holm correction, patients with incomplete RTW had significantly lower scores on the TMT-B, Stroop Color-Word, and DEX-total compared to patients with complete RTW, with moderate to large effect sizes.

The following variables were included in block 1 of the binary logistic regression analysis with as dependent variable RTW: age, educational level, SAH-type, and external CSF drainage (Table 4). A test for the constant only against the full model was not significant, indicating that RTW could not be reliably predicted by a model including these predictors (χ^2^ = 13.05, *p* = 0.11, Nagelkerke R^2^ = 0.23).

**Table 4:** Results of binary logistic regression on RTW.

		95% CI for Odds Ratio		
	B (SE)	Lower	Odds Ratio	Upper
***Included in Block 1***				
**Age**	-0.009 (0.04)	0.92	0.99	1.07
**Educational level (5)**	1.90 (1.36)	0.47	6.71	96.21
**SAH type (1)**	-0.29 (0.61)	0.41	1.34	4.43
**External drainage (1)**	-1.36 (0.57)*	0.08	0.26	0.78
***Included in Block 2***				
**Age**	0.01 (0.05)	0.92	1.01	1.10
**Educational level (5)**	0.82 (1.48)	0.12	2.27	41.42
**SAH type (1)**	-0.07 (0.82)	0.19	0.94	4.67
**External CSF drainage (1)**	-1.40 (0.67)*	0.07	0.25	0.92
**Stroop Color-Word**	0.008 (0.02)	0.97	1.01	1.05
**TMT-B**	0.01 (0.01)	0.98	1.01	1.04
**DEX**	0.10 (0.04)*	1.02	1.10	1.20

RTW, return to work; SAH, subarachnoid hemorrhage; TMT, Trail Making Test. Coding Educational level: 5 = university; SAH type: 1 = anSAH; external CSF drainage: 1 = external CSF drainage.

* *p* < 0.05

In block 2, the neuropsychological measures for which significant differences were found between patients with incomplete and complete RTW were added to the model: TMT-B, Stroop Color-Word, and DEX-total. The block chi-square statistic was significant (χ^2^ = 10.63, *p* = 0.01), indicating that Model 2 was superior to the first model in terms of overall model fit. A test of the full model against a constant only model was also statistically significant (χ^2^ = 23.68, *p =* 0.01, Nagelkerke R^2^ = 0.39), indicating that the predictors as a set reliably distinguished between complete and incomplete RTW. With this model, 81.7% of the patients were correctly classified as having incomplete or complete RTW. Incomplete RTW was correctly classified in 89.4% of all cases.

The only statistically significant predictors in the final model were external CSF drainage and DEX-total (Table 4). Patients with higher scores on the DEX, indicating executive impairment in daily life, are slightly more likely to have incomplete RTW. The odds ratio for external CSF drainage indicates that as drainage changes from no drainage (0) to drainage (1), the change in odds of having incomplete RTW compared to complete RTW is 0.25. In other words, the odds of a patient with external drainage having incomplete RTW are 1/0.25 = 4 times more than for a patient without external drainage.

## Discussion

This study shows that poor performances on tests for complex attention and executive functions were related to incomplete long-term RTW post-SAH. Furthermore, neuropsychological measures, demographic and SAH-related variables together were predictors for incomplete RTW, while demographic and SAH-related variables alone were not.

Only one-third of all SAH patients in this study reported complete work resumption, consistent with previous studies on post-SAH RTW [1,3,6]. This is the first study that examined the relationship between a core set of cognitive functions and RTW, in both aSAH and anSAH patients. We found lower scores on measures for complex attention and executive functions in patients with incomplete RTW compared to patients with complete RTW. In previous studies, scores on a cognitive screening tool and RTW were found to be significantly related to each other [22,23]. Although a screening tool is easy to apply, it does not allow for distinction between different cognitive domains and has low specificity compared to a comprehensive neuropsychological battery [45]. Among the cognitive functions investigated in the present study, complex attention and executive functions appear to be specifically important with respect to RTW. This is not surprising, since these so called higher-order functions comprise those mental capacities needed to initiate, monitor, and regulate complex behavior [44], which allow us to adapt to new situations. Interestingly, patients with incomplete RTW particularly performed worse on tasks that had to be completed as quickly as possible. One could imagine that time pressure is an important factor for several work situations, when fast decision making and finishing certain tasks within working hours are crucial.

In our study, RTW could not be significantly predicted using only demographic characteristics and acute SAH-related variables. Importantly, including measures of complex attention and executive functions, SAH-related variables and demographic characteristics, resulted in a prognostic model that could reliably distinguish between complete and incomplete RTW. Incomplete RTW could be correctly classified in almost 90% of all cases. Of the included neuropsychological measures, it was only the self-reported executive impairment in daily life that emerged as a significant predictor of incomplete RTW. Although tests are important to assess cognitive functions objectively, they may not always take the full range of problems and experienced burden into account [46]. Consequently, self-report measures of cognitive problems may represent a different perspective on these problems that is important to take into account.

Considering acute SAH-related variables in the final model, the odds of patients with external CSF drainage having incomplete RTW were higher than for patients without drainage. Previous studies indicated an association between external CSF drainage and impaired functional outcome [47] and poor quality of life [48]. However, the relationship between external CSF drainage and RTW has not been previously described. Furthermore, SAH-type showed no predictive value with respect to RTW in the model. This might indicate that RTW is affected by (sub)acute factors in the same manner after both aSAH and anSAH. Alternatively, one could speculate that this finding is due to different rates of external CSF drainage, with a significantly higher rate of external CSF drainage in aSAH patients. Additionally, neither age nor education were identified as significant predictors in the final model of RTW in the long-term, in contrast to previous findings by Vilkki and colleagues[11]. A possible explanation for this might be distinct measurement methods of RTW (a structured interview, whereas Vilkki et al. used a questionnaire).

Some limitations of this study should be mentioned. Firstly, our results are only applicable to previously employed patients. One could imagine that cognitive functioning is also related to other aspects of everyday life functioning, such as social participation. For example, emotion recognition deficits have been associated with impaired psychosocial functioning in stroke and traumatic brain injury populations [41,49]. Future research might explore this relationship, in order to expand findings to unemployed SAH patients. Also, working environment and therefore return to work possibly differs between countries or cultures, so our results do not necessarily apply to other regions of the world. Secondly, neuropsychological assessment, although providing important information about cognition, may not be applicable in all patients after SAH, because of poor clinical condition. Although severity in our patient group varied, most patients had a relatively good initial clinical condition. Therefore, the present findings might not be generalizable to more severely impaired SAH patients and might underestimate the problems with RTW and cognitive impairments. Lastly, RTW is a multifaceted construct, likely to be influenced by a lot of different variables. Unfortunately, although considerable for SAH research, our sample size was too small to analyze all possible factors influencing RTW. However, our main goal was to investigate the additional predictive value of several cognitive functions for RTW, to improve the identification of those patients who are at-risk for incomplete RTW.

Concluding, these results strongly suggest that cognitive functions, specifically complex attention and executive functions, that can be measured in the subacute stage post-SAH substantially contribute to long-term RTW. Moreover, early identification of patients at risk for incomplete RTW seems insufficient if this is based on only SAH-related and demographic variables, collected in the acute stage. Long-term RTW of SAH patients was best predicted by adding complex attention and executive functions as measured in the subacute stage. Our findings indicate the need for the incorporation of neuropsychological assessment in regular follow-up of both aSAH and anSAH patients. This enhances our insight into patients’ strengths and deficits, can be beneficial to timely identification of those patients at risk for not returning to work, and in creating early interventions to ultimately improve long-term RTW.
